# The measurement of binding affinities by NMR chemical shift perturbation

**DOI:** 10.1007/s10858-022-00402-3

**Published:** 2022-08-03

**Authors:** Billy Hobbs, Jack Drant, Mike P. Williamson

**Affiliations:** 1 School of Biosciences, University of Sheffield, Firth Court, Western Bank, Sheffield, S10 2TN UK; 2 Present Address: Astbury Centre for Structural Molecular Biology, University of Leeds, Leeds, LS2 9JT UK; 3 Present Address: Department of Chemistry, University of Warwick, Coventry, CV4 7AL UK

**Keywords:** Affinity, Binding, Dissociation constant, NMR, Chemical shift, Conformational change

## Abstract

We have carried out chemical shift perturbation titrations on three contrasting proteins. The resulting chemical shifts have been analysed to determine the best way to fit the data, and it is concluded that a simultaneous fitting of all raw shift data to a single dissociation constant is both the most accurate and the most precise method. It is shown that the optimal weighting of ^15^N chemical shifts to ^1^H chemical shifts is protein dependent, but is around the consensus value of 0.14. We show that chemical shift changes of individual residues can be fit to give residue-specific affinities. Residues with affinities significantly stronger than average are found in close contact with the ligand and are suggested to form a rigid contact surface, but only when the binding involves little conformational change. This observation may be of value in analysing binding and conformational change.

**Supplementary Information:**

The online version contains supplementary material available at 10.1007/s10858-022-00402-3.

## Introduction

An important application of biomolecular NMR is to study the binding of ligands to proteins. A common way of doing this is to label the protein with ^15^N, and conduct a series of HSQC experiments in which a ligand (which could be a small molecule or a macromolecule) is titrated gradually into the protein (Zuiderweg 2002). Given suitable controls, changes in chemical shift or intensity in the signals of the target protein can be interpreted as indicating binding. The residues that change most are assumed to represent the binding site, and the chemical shift (or intensity) changes can often be fitted to a binding curve to obtain affinities, characterised by the dissociation constant *K*_d_(Williamson 2013). This technique has been applied widely. Despite the widespread use of this method, there has (to our knowledge) never been any systematic study of the most appropriate way to fit the chemical shift changes observed in the HSQC spectra. Therefore in this work, we have studied three protein/ligand interactions with different biological properties, and conducted tests aimed at determining the best way to analyse the results. We show that the most reliable results are obtained by fitting all the shifts together, and that the affinities resulting from this method are considerably more precise and slightly stronger than those obtained by selecting peaks and fitting them. A statistical analysis of the data indicates that the *K*_d_ values obtained from different amino acid residues are genuinely different: in other words, there is not a single affinity but many. The differences may allow a distinction between the active site and the rest of the protein and may provide a measure of the extent of conformational change on binding.

## Materials and methods

### Protein expression and purification

Three proteins were selected for study. Lysostaphin is a bacteriolytic enzyme produced by *Staphylococcus simulans* biovar *staphylolyticus* and contains a catalytic domain and a domain that binds to bacterial peptidoglycan, described as SH3b (Thumm and Götz 1997). The SH3b domain (93 residues) was expressed and purified as described (Gonzalez-Delgado et al. 2020). The gene was expressed in *E. coli* BL21(DE3) cells in a pET15b vector with a His-tag. It was purified on a nickel column followed by gel filtration on a Superdex S75 column, and concentrated into 50 mM sodium phosphate pH 6. The YG_5_ peptide ligand (the hexapeptide YGGGGG) was synthesised by Peptide Protein Research.

Barnase (110 residues) is a well-characterised RNase from *Bacillus amyloliquefaciens*. The catalytically inactive H102A mutant of barnase (Mossakowska et al. 1989) was expressed and purified as described (Cioffi et al. 2009). Briefly, the barnase gene was carried on the pQE-60 plasmid and transformed into *Escherichia coli* M15 [pREP4] cells. It was expressed in M9 medium containing ampicillin, kanamycin and ^15^N ammonium chloride, induced using 1 mM IPTG, and purified using a Q-sepharose column followed by a SP sepharose column. Protein was exchanged into 50 mM sodium acetate, 2 mM sodium azide, pH 5.8 using a Vivaspin. The ligand for barnase was d(CGAC), synthesised by Metabion International AG (Martinsried, Germany) and used without further purification.

HisJ (241 residues) is a periplasmic binding protein from *E. coli* which binds histidine and brings it to inner membrane transporters (Oh et al. 1994). It undergoes a large conformational change on binding and closes around the ligand (Felder et al. 1999). It was contained on a pET22b plasmid and transformed into *E. coli* BL21(DE3) cells. Cells were grown in ^15^N-labelled M9 medium, induced with 0.5 mM IPTG, and sonicated. HisJ was purified by ammonium sulfate precipitation, using the supernatant from 60% saturation, and then purified on a Superdex 75 gel filtration column. Residual bound histidine was removed by denaturing the protein using 4 M guanidinium hydrochloride and then refolding by dialysis. The protein was concentrated into 50 mM sodium phosphate, 2 mM sodium azide, pH 7.4 using a Vivaspin. The ligand used was lysine (Sigma Aldrich).

### NMR titrations

All titrations were planned to give a good quality set of data: ideally going up to a 20-fold excess of ligand over protein, with high ligand stock concentration (to avoid unnecessary dilution of the protein) and approximately 13 titration steps, with a greater density of points near the start of the titration to be able to define the shape of the binding curve well. In all cases, the ligand was prepared in identical buffer to the protein, and pHs of protein and ligand solutions carefully adjusted to be identical. Concentrations of ligand and protein were determined using 1D NMR with 10 s relaxation delay, using 1 mM DSS as an internal standard. Preliminary experiments showed that measurement (and adjustment where necessary) of pH at each titration point gives poor results, due to loss of sample and unpredictable pH variation. We therefore simply added ligand directly to the NMR tube at each titration, and measured the pH at start and end of the titration. The pH variation over the course of the titrations when carried out in this way was less than 0.1 pH units. For the SH3b/YG_5_ titration, we used a ligand stock concentration of 1.7 mM for the first 3 additions (in order to pipette the ligand volume sufficiently accurately), and thereafter a stock of 15 mM. The initial protein concentration was 50 μM, with 13 titration points, and the ratio of ligand to protein at the final titration point was 102. This high ratio was used because the affinity was weak and therefore we needed a higher final ligand ratio to get close to saturation. For the barnase/d(CGAC) titration, the ligand stock was 5 mM, initial protein concentration 50 μM, 14 titration points, and the ratio of ligand to protein at the final titration point was 19. For the HisJ/lysine titration, the lysine stock was 5 mM, initial protein concentration 46 μM, 14 titration points, and the ratio of ligand to protein at the final titration point was 21. All titrations were obtained on a Bruker DRX-600 spectrometer with a cryoprobe at 298 K, using 5 mm tubes. HSQC experiments used the standard sensitivity enhanced Bruker pulse program hsqcetfpf3gpsi. For HisJ, TROSY spectra were used as they had sharper signals. The sample was equilibrated for at least 15 min before each titration to allow the temperature to settle down. All measurements were made at 25°C and used a spectral width of 16 ppm for ^1^H and 36 ppm for ^15^N, with maximum acquisition times of 0.106 s and 0.058 s respectively. Spectra were processed using a cosine bell squared function in both dimensions. The sequence-specific assignments of the HSQC spectra were copied from known assignments in the BioMagResBank (Ulrich et al. 2008), and were checked using 3D spectra using double labelled protein in cases of ambiguity.

### Data analysis

Spectra were processed in Topspin and referenced to internal DSS. The spectra were then transferred to Felix (Felix NMR, Inc., San Diego, CA) for peak picking and assignment. Initial data fitting was carried out using locally written scripts that carried out least-squares fitting using a Levenberg–Marquardt algorithm. This method was used for fitting of individual nuclei. Subsequent global fittings were carried out using RStudio 4.0.2. Individual fittings were also repeated using RStudio, with essentially identical results. All titration data were fitted to the standard equation for a 1:1 binding equilibrium (Williamson 2013):

1 $$\Delta \delta_{{{\text{obs}}}} = \Delta \delta_{{{\text{max}}}} \left\{ {\left( {\left[ P \right]_{{\text{t}}} + \, \left[ L \right]_{{\text{t}}} + K_{{\text{d}}} } \right) \, {-} \, \left[ ( \right[P\left] {_{{\text{t}}} + \, } \right[L\left] {_{{\text{t}}} + K_{{\text{d}}} )^{{2}} - {4}} \right[P\left] {_{{\text{t}}} } \right[L\left] {_{{\text{t}}} } \right]^{{{1}/{2}}} } \right\}/{2}\left[ P \right]_{{\text{t}}}$$

To obtain the “combined” chemical shift change for each amino acid from the separate ^1^H and ^15^ N nuclei, shifts were combined according to

2 $$\delta _{{comb}} = \sqrt {{\raise0.7ex\hbox{${\left( {\delta _{H} ^{2} + \left[ {\alpha \delta _{N} } \right]^{2} } \right)}$} \!\mathord{\left/ {\vphantom {{\left( {\delta _{H} ^{2} + \left[ {\alpha \delta _{N} } \right]^{2} } \right)} 2}}\right.\kern-\nulldelimiterspace} \!\lower0.7ex\hbox{$2$}}}$$

where α is a weighting factor discussed in the text. Protein structures were visualised using Pymol (Schrödinger, Inc.). The residue numbering used here follows the numbering used for the NMR assignments and in the Supplementary Tables. By comparison to the crystal structures, this means that residue numbering for SH3b is 399 less; barnase is the same; and HisJ is 3 more.

## Results

### Survey of chemical shift changes

The aim of this study was to take three proteins with different functions, and therefore hopefully contrasting ligand binding modes, and analyse ligand binding data using ^15^N HSQC titrations. The proteins should be stable, monomeric, bind one equivalent of ligand at or close to fast exchange conditions, have assigned NMR spectra, and bind tightly enough that we could get close to full binding saturation without excessive amounts of ligand. We therefore selected the following:

1. SH3b is a domain from lysostaphin, which has been characterised in detail (Gonzalez-Delgado et al. 2020). It binds peptides found in bacterial cell walls, and the function of the binding is thought to be to localise the hydrolytic domain of lysostaphin close to its peptidoglycan substrate: that is, it functions purely as a binding domain. It has two different binding sites: one for the peptide stem, and one for the pentaglycine crossbridge typical of *Staphylococcus aureus (*Schleifer and Kandler 1972). The binding site for pentaglycine is a tight and rigid groove, and there appears to be little structural change to the protein when it binds (Gonzalez-Delgado et al. 2020). In this work, we used the ligand YGGGGG, which was chosen because the pentaglycine part fits very neatly into the groove in the protein, while the N-terminal tyrosine is not part of the native ligand and is expected to bind in a much more flexible manner, while producing measureable chemical shift changes due to the ring current from the tyrosine ring.
2. Barnase is a bacterial RNase. There is a crystal structure of free protein and of the complex with d(CGAC) (Buckle and Fersht 1994), and NMR studies have confirmed that the deoxynucleotide substrate analogue d(CGAC) binds to the active site (Cioffi et al. 2009), and that there are only small structural changes on binding, corresponding to a hinge closure plus the closing of a “lip” forming a ring around the edge of the active site (Pandya et al. 2018). The residues forming the active site are well characterised by the crystal structure.
3. HisJ is a periplasmic binding protein, whose function is to bind histidine in the periplasm and transport it to an ABC transporter. It has two lobes which fold around the ligand (Oh et al. 1994), creating a major conformational change on binding in what has been described as a Venus flytrap motion (Felder et al. 1999). The binding of histidine is in slow exchange on the NMR timescale (Paul et al. 2017), and so we used lysine as the ligand, which binds more weakly and is in fast exchange.

**Fig. 1:**
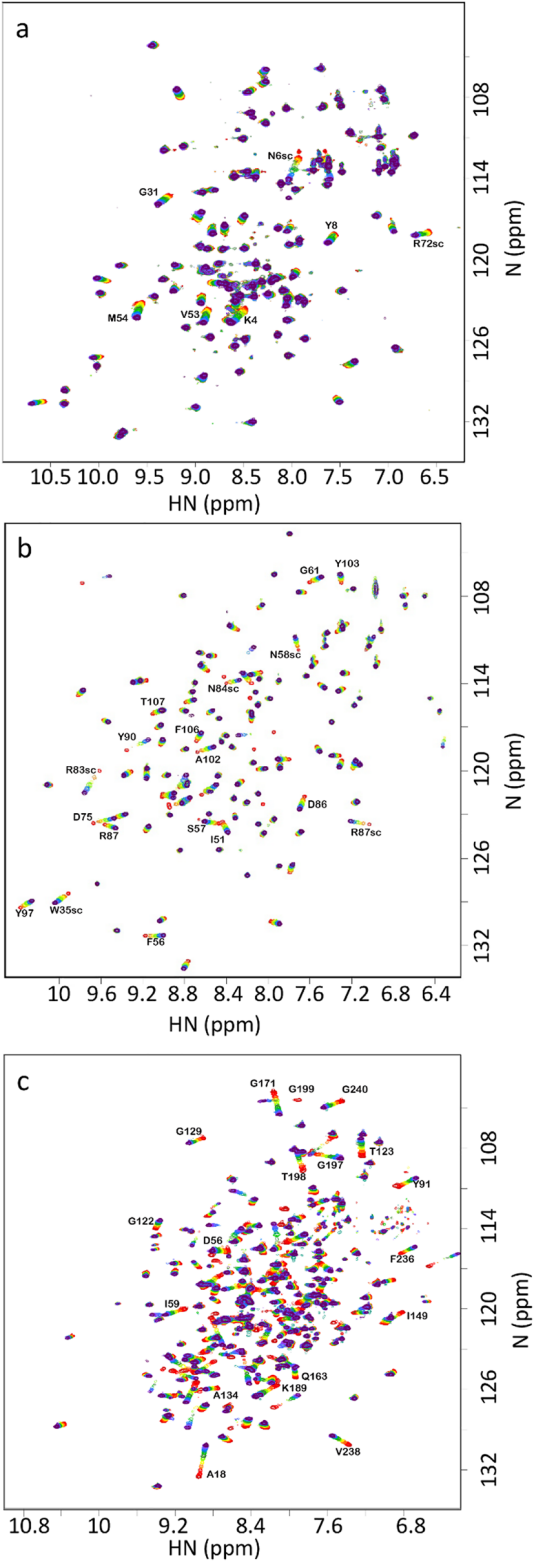
^15^N HSQC titration data for **a** SH3b **b** Barnase **c** HisJ. In each case, peaks are colored from red to violet with addition of ligand. Signals undergoing large shift changes are labelled

**Fig. 2:**
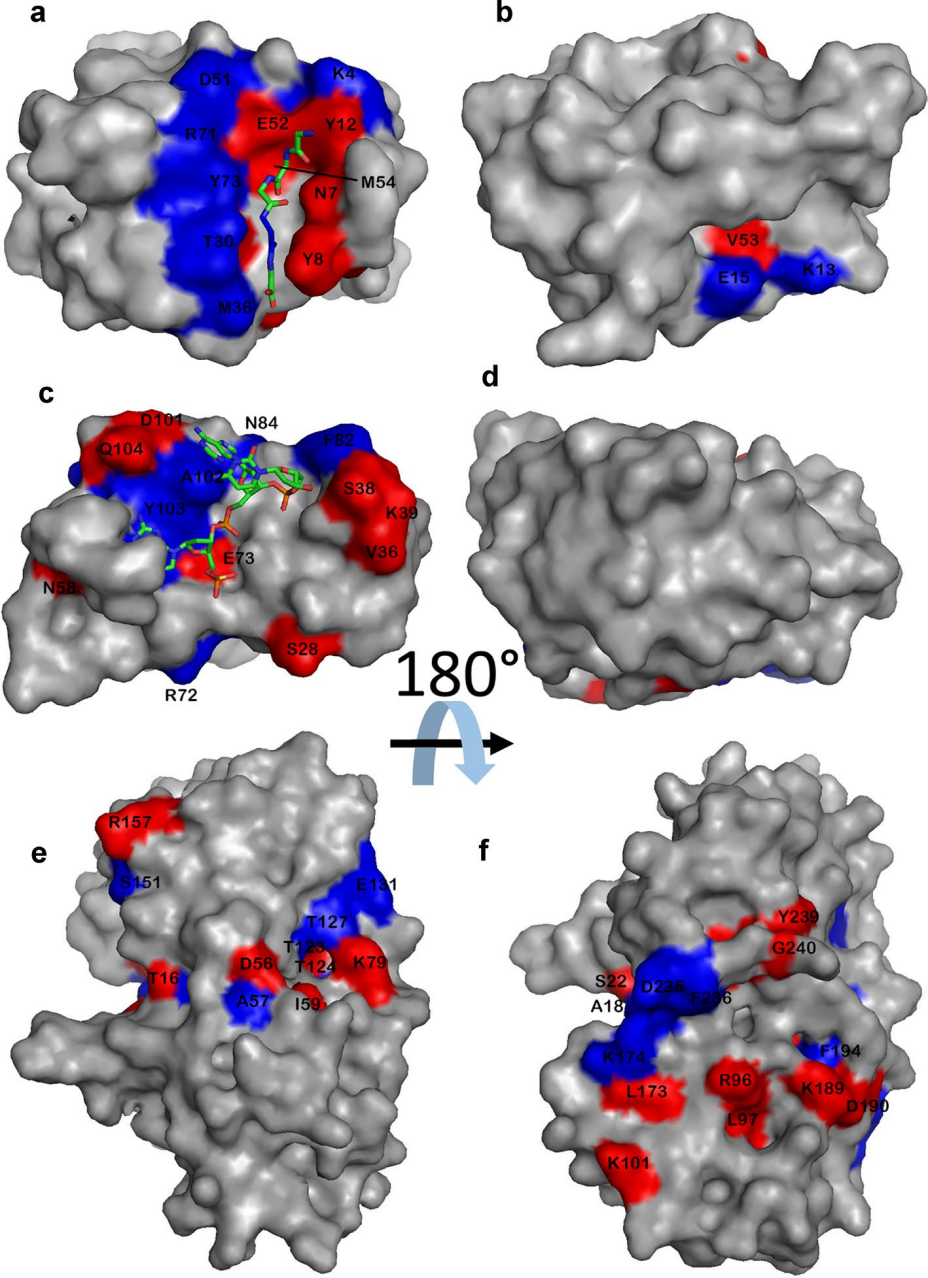
The locations of large chemical shift changes on titration of **a**, **b** SH3b **c**, **d** barnase **e**, **f** HisJ with their ligands. In each case, the right panel is a rotation of 180° around a horizontal axis. Blue is used to denote negative (upfield) chemical shift changes in H or N, and red for positive (downfield) changes. For SH3b and barnase, the ligand is denoted by sticks. For HisJ the ligand is completely buried and is in the center of the protein. The shift changes shown include approximately 20% of the amino acids in the protein that have reliably fitted shift changes. Residues undergoing large shift changes are labelled

Each protein was expressed in ^15^N-labelled M9 medium and purified. NMR assignments were taken from published data and confirmed by triple resonance experiments where necessary. The proteins were each titrated with ligand. HSQC spectra are shown in Fig. 1, indicating high purity of the proteins, and good quality titrations showing linear chemical shift changes in the HSQC spectrum, as expected for a simple 1:1 binding in fast exchange (Williamson 2013). The locations of chemical shift changes due to ligand binding are shown in Fig. 2. For SH3b, the chemical shift changes affect only a small number of residues (Fig. 1a), which are clustered around the binding site. They form a striking pattern, with residues within the groove having positive chemical shift changes and residues lining the sides of the groove having negative shift changes (Fig. 2a). Changes in shift of ^1^H and ^15^N have similar distributions, implying that it makes sense to consider both together. For barnase, shift changes are less tightly grouped around the binding site, but the majority of shifted residues are still close to the ligand (Fig. 2c, d). Shift changes are more numerous and more widespread for HisJ, as expected because of its larger conformational change on binding (Fig. 2e, f). The shift changes therefore behave as expected: SH3b binds with little conformational change, and has shift changes closely localised to the binding site, while in contrast HisJ has a large-scale hinge bending motion and has shift changes more widely spread around the protein surface.

**Fig. 3:**
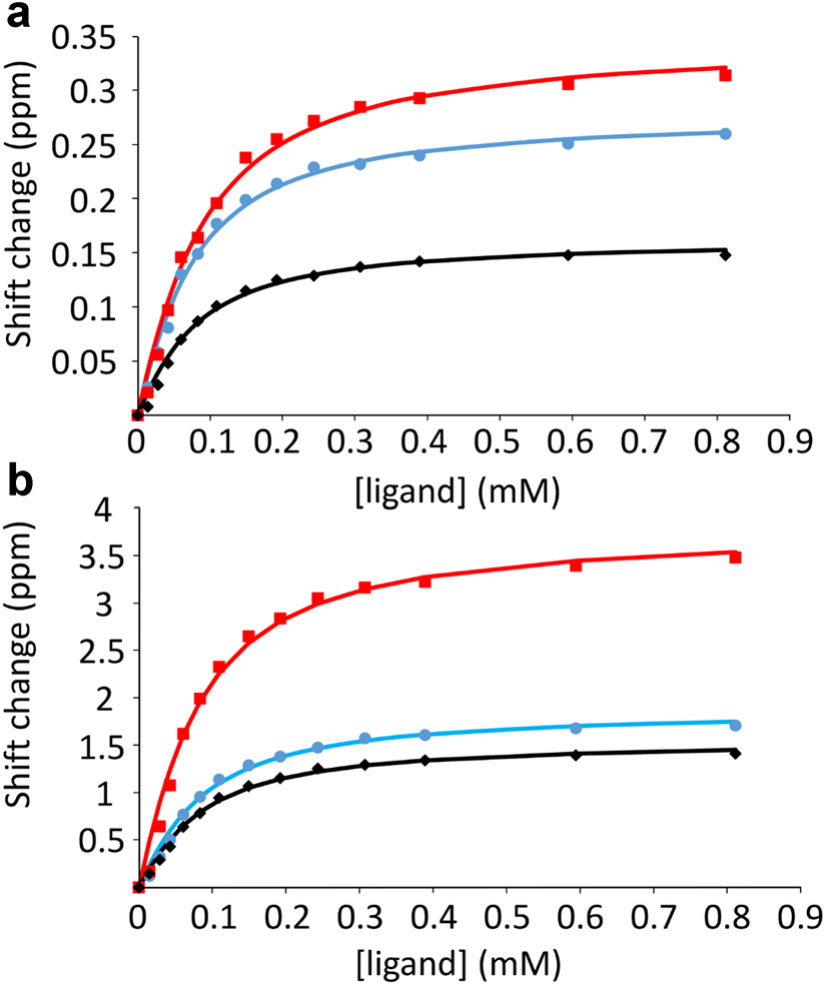
Typical chemical shift titration data: HisJ, showing change in chemical shift with addition of the ligand lysine. The curves are individual best fits to the data. **a** Shift changes in ^1^H. Data are shown for T77 (red: fitted to *K*_d_ of 62 ± 6 μM), D14 (blue: fitted to *K*_d_ of 49 ± 6 μM), and G129 (black, fitted to *K*_d_ of 54 ± 12 μM). **b** Shift changes in ^15^N. Data are shown for F194 (red: fitted to *K*_d_ of 56 ± 5 μM), G171 (blue: fitted to *K*_d_ of 60 ± 11 μM), and T198 (black: fitted to *K*_d_ of 58 ± 13 μM). All data are shown as positive shift changes for ease of presentation. The actual shift changes for T77, D14, F194 and T198 are negative

The chemical shift changes observed follow the shape of a standard saturation curve for all three proteins, suggesting that it should be possible to fit good *K*_d_ values (Fig. 3).

### Fitting individual shift changes to obtain *K*_d_ gives large variation

**Fig. 4:**
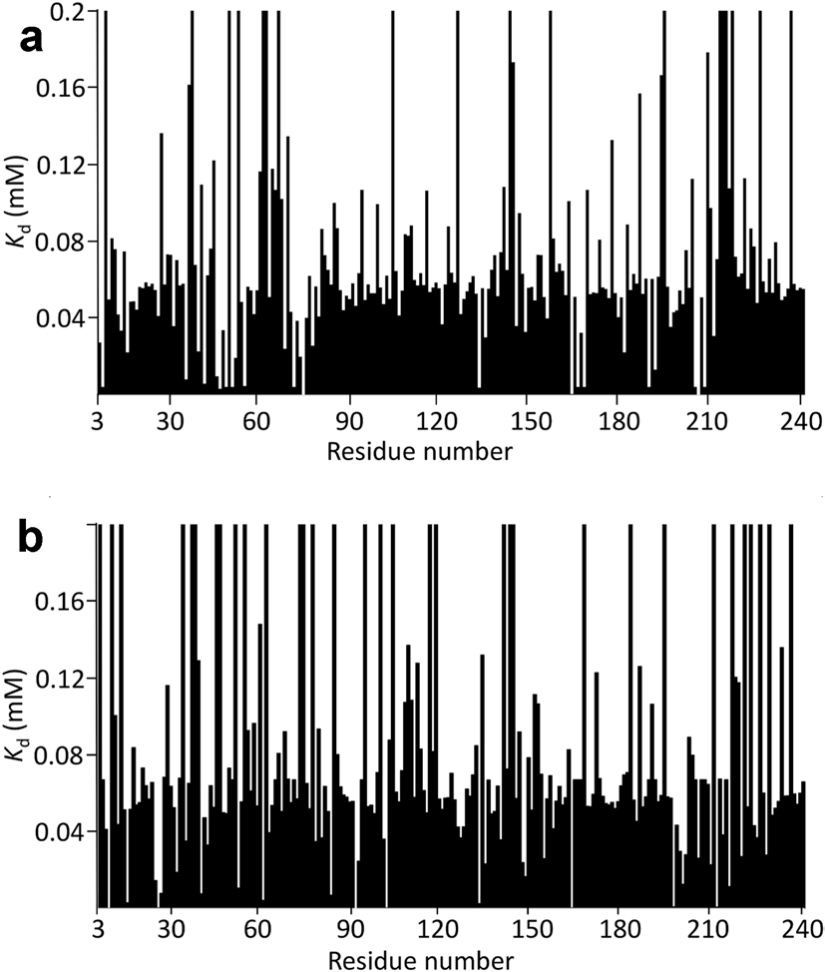
*K*_d_ values fitted for HisJ binding to lysine, for **a** ^1^H **b** ^15^N. Data are shown for all residues that could be fitted. A small number are not shown, mainly because of overlap or because they are prolines. Note that the *K*_d_ values are truncated at 0.2 mM: many of the truncated residues are much larger than this

The most obvious way to estimate *K*_d_ from NMR data is to measure the chemical shifts for each nucleus, fit a selection of nuclei individually, and then average the resulting values. However, this method produces inaccurate and imprecise values, as we now show. Typical results (for HisJ) using this method are shown in Fig. 4; data for barnase and SH3b are in SI. There is a wide variation in fitted values. Many of the fitted values for HisJ cluster around 50–60 μM, but there are many residues with much larger (weaker) *K*_d_ values, and several with much smaller (stronger) values. If one just takes all the fitted values and calculates the mean it comes out to 4.8 ± 2.1 mM for ^1^H and 37 ± 23 mM for ^15^N, which are values that are dominated by a few very large fitted *K*_d_ values and clearly do not represent the true affinity (Fig. 4). [Note that here and subsequently, the error values quoted are standard errors.] On examination of the individual fits it is clear that most of the large fitted values derive from chemical shift changes that are small and nearly linear, and often fit to unfeasibly large shift changes at saturation, Δδ_max_. These may arise from very weak secondary binding, or possibly from effects of the ligand on solvent structure, sensed by the protein as small and almost linear shift changes (Bye et al. 2016). They may also derive from small changes in pH during the titration. Most of the small fitted *K*_d_ values derive from residues that have a small chemical shift change at the first addition of ligand but then do not shift thereafter. These are widespread phenomena and do not represent genuine site-specific binding in either case. It therefore seems reasonable to exclude such data.

**Fig. 5:**
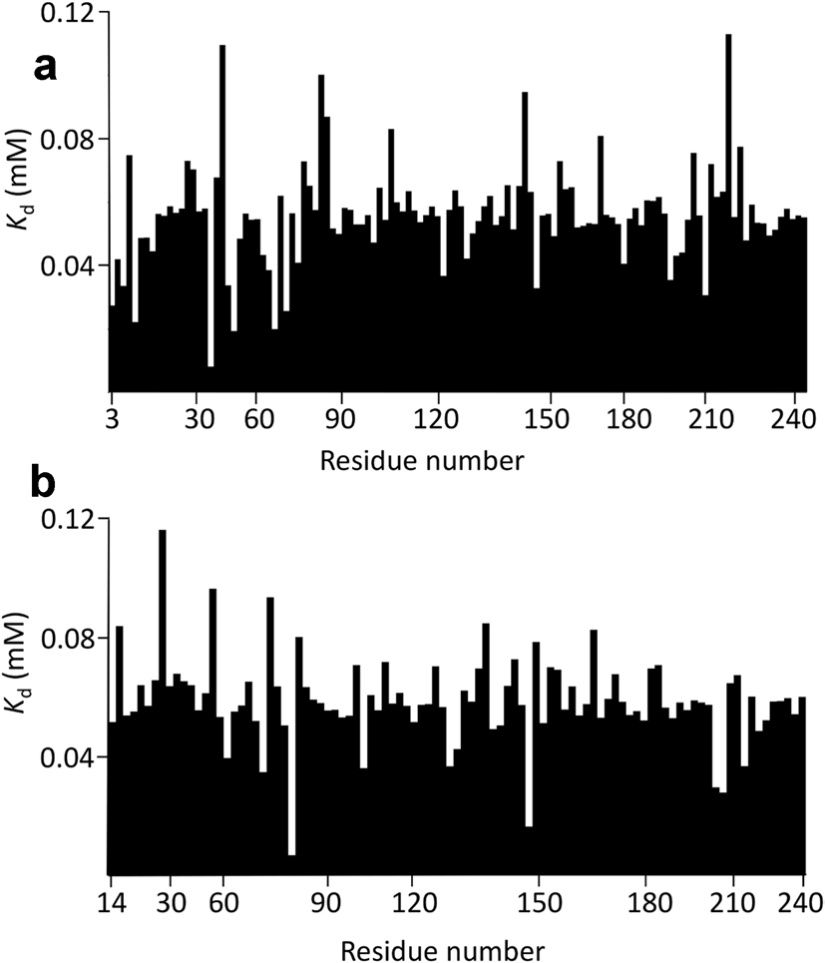
*K*_d_ values fitted for HisJ binding to lysine, after filtering out unreliable values, for **a** ^1^H **b** ^15^N. Nuclei were removed if they had a fitted *K*_d_ of > 200 μM, total ^1^H shift changes of < 0.03 ppm, or total ^15^N changes of < 0.15 ppm. It was not necessary to filter out very small *K*_d_ values because these were removed by the chemical shift limits

We conclude that if affinities are to be estimated from averaging over individual fits, then some kind of selection criteria need to be imposed. Following the arguments above, the obvious filtering is to exclude fitted *K*_d_ values that are either very much larger or very much smaller than the consensus; and exclude residues for which the total chemical shift change during the titration is very small, such that any chemical shift changes measured are unreliable. Carrying out such filtering results in exclusion of about half of the data, and gives much better results (Fig. 5), now with a fitted *K*_d_ of 57.9 ± 2.3 μM for ^1^H and 62.5 ± 3.8 μM for ^15^N, which are identical within error, and much more reasonable values. Similar results (but different affinities) were found for SH3b and barnase (Table 1 and supplementary information).

**Table 1:** *K*_d_ values (μM) fitted using different methods

Method*	Nucleus	SH3b	Barnase	HisJ
Individual	H	329 ± 30	36.3 ± 0.8	57.9 ± 2.3
	N	295 ± 17	35.9 ± 0.8	62.5 ± 3.8
	Combined	312 ± 17	36.1 ± 0.6	60.0 ± 2.1
By nucleus	H	281 ± 4	35.5 ± 0.5	54.3 ± 0.6
	N	307 ± 4	35.3 ± 0.5	56.1 ± 0.5
	Combined	294 ± 13	35.4 ± 0.1	55.2 ± 0.9
All		296 ± 3	35.4 ± 0.4	55.3 ± 0.4

*Individual means that each nucleus was fitted separately (after filtering out unreasonable fits, as shown in Fig. 5), and then values were averaged together to give the mean and standard error shown. *H*: values obtained by fitting only ^1^H shifts. *N*: values obtained by fitting only ^15^N shifts. *Combined*: H and N shifts for each amino acid were combined together following Eq. (2) before averaging. *By nucleus*: all H nuclei and all N nuclei were fitted together. The *combined* figure is obtained by simply averaging these two results. *All*: all nuclei were fitted together to a single value of *K*_d_

### Best fits result from fitting all shifts together

One feels that fitting each nucleus separately and then averaging is an unsatisfactory method; partly because of the arbitrary selection of *K*_d_ limits and shift changes, as indicated in the legend to Fig. 5; and partly because statistical theory suggests that better estimates of the population can be obtained by fitting all data simultaneously, with the resulting error in the fitted value being proportional to $1/\sqrt{n}$. One therefore wants to maximise *n*, the number of data points fitted. We therefore fitted all shift changes simultaneously to a single *K*_d_ value. The results are given in Table 1.

Inspection of the data in Table 1 shows that the resulting fitted *K*_d_ values are not strongly dependent on the fitting method. One would expect that the chemical shift changes of an amide proton should measure the same *K*_d_ as the chemical shift changes of the attached nitrogen: that is, that fitting H or N or the combined shift (following Eq. (2)) should all produce the same affinity. This is true: for H and N pairs, the differences are not significant at *p* < 0.05 according to a Student’s *t*-test. Unsurprisingly, fitting to the combined shift gives a value intermediate between the H and N values, although the error is not necessarily lower. A single fitting of all nuclei together to a single *K*_d_ gives the smallest error. We note that for all three proteins, fitting of individual shift changes produces a larger value of *K*_d_ than fitting all nuclei together, presumably from the same effect as discussed above, that the fitting of selected individual residues includes some residues with unreasonably large *K*_d_ values. In other words, the data shown here suggest that fitting of individual nuclei and then averaging tends to produce a systematically weaker affinity than the correct value, although one that remains within the larger error limits resulting from such a fit; conversely, fitting all nuclei together to a single affinity results in both a more precise and a more accurate value.

Our datasets allow us to determine the optimum value for each protein of α, the weighting of N shifts vs H shifts (when measured in ppm) to assign equal overall weights to both nuclei. Here, α was determined by fitting the maximum chemical shift change on saturation (Δδ_max_ from Eq. (1)) for each pair of nuclei, and finding the ratio between them. The values of α found were 0.14 for SH3b, 0.20 for barnase and 0.21 for HisJ, suggesting that the consensus value of 0.14 (Williamson 2013) is a reasonable value, though specific for each protein.

### Each residue has a different *K*_d_

**Fig. 6:**
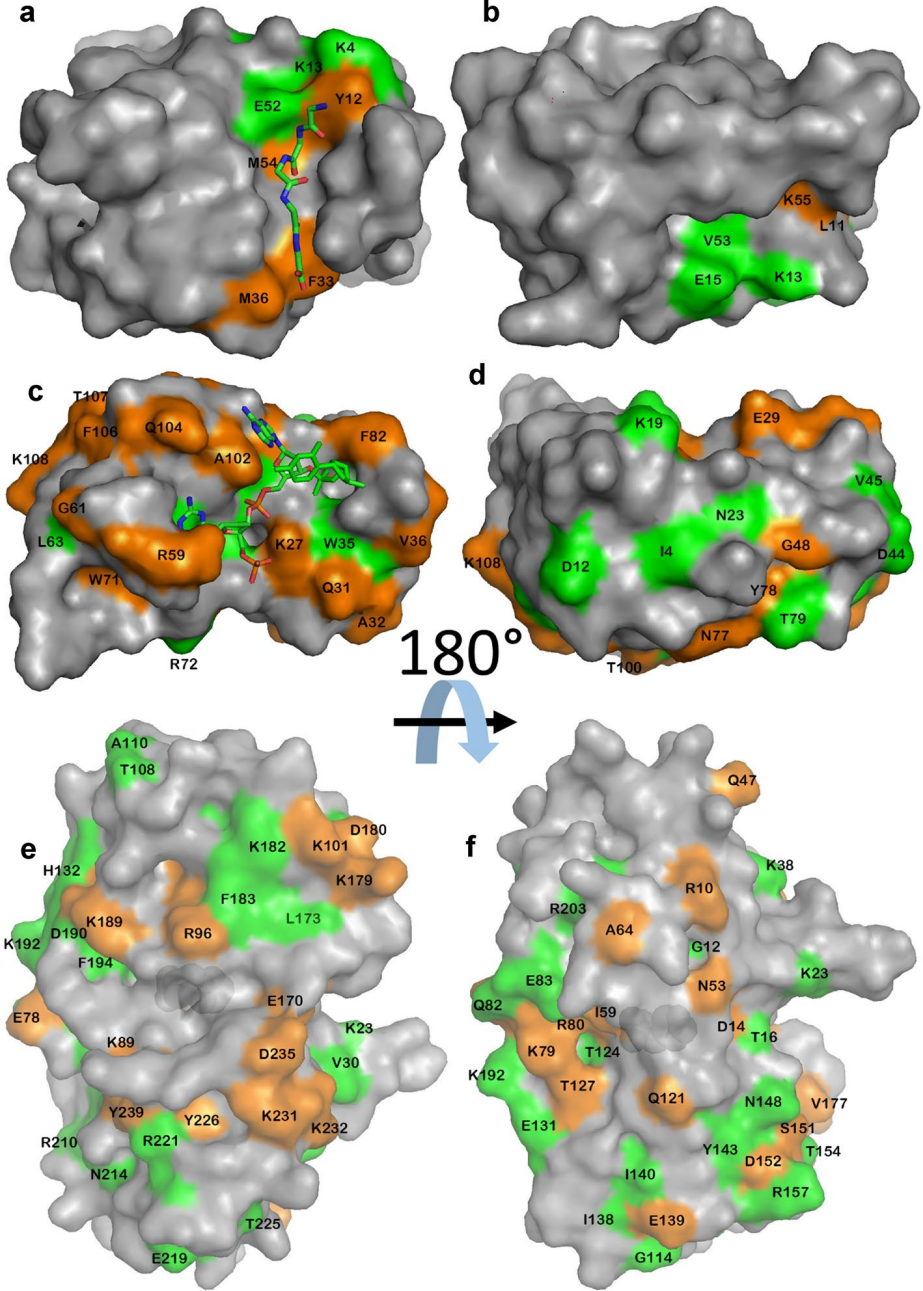
Residues fitting to *K*_d_ values significantly different from the mean (defined as outside the 99% confidence intervals), for **a** SH3b **b** barnase, **c** HisJ. Residues with *K*_d_ values significantly smaller (stronger) than the mean are in orange, and residues significantly larger (weaker) are in green, and are labelled. In each case, the right panel is a rotation of 180° around a horizontal axis. For SH3b and barnase the ligand is shown as sticks; for HisJ the protein surface is drawn slightly transparent, allowing the bound histidine ligand to be seen in the center of the protein. For SH3b the ligand shown is GGGGG, using the crystal structure with PDB ID 5leo. The ligand used here is YGGGGG, where the additional tyrosine is at the N-terminal (top, in the view shown in **a**) end of the peptide

We have already noted in Figs. 3, 4, 5 the spread of values seen for *K*_d_ when fitted to individual nuclei. Is this real, and if so, does this variation have any meaning? To address this, a likelihood ratio test was carried out, which showed a highly significant improvement in fit when fitting individual shifts compared to fitting for a single global *K*_d_, even allowing for the fact that very many more individual variables were fitted in the first case. In other words, statistics suggests that there is a genuine variation in *K*_d_ across the protein. For each protein, we therefore went back to the residue-specific fits, and identified residues that have a *K*_d_ value which is likely to be significantly different from the average. This was done by calculating a 99% confidence interval for the *K*_d_, given by mean ± *z*σ/$\sqrt{n}$, where σ is the standard deviation of the fitted *K*_d_ values, *n* is the number of values, and *z* is 2.58 for a 99% confidence. This calculation was done separately for ^1^H and ^15^N nuclei, as an internal control. Residues with values outside this range potentially do have affinities genuinely different from the mean. However, on inspection of the data it was clear that there remain a few residues with very extreme fitted affinities. We noted above that it is possible to fit very weak affinities when the titration data are almost linear; and very strong affinities when the shifts change only at the first titration point. It therefore seemed prudent to exclude such fits, and thus to remove any residues with a fitted *K*_d_ value more than 2σ from the mean, as these are likely to be erroneous values. The resulting residues are identified in Fig. 6. Almost half of the residues in each protein are found in this “significantly different affinity” group, this being a considerably larger fraction than would be expected for a normal distribution, providing further evidence that there is a genuine range of *K*_d_ values. There is a reasonably good agreement between residues identified in the two sets of nuclei, increasing our confidence that the variation in *K*_d_ is meaningful.

For SH3b (Fig. 6a, b) the results are clear and striking: most of the residues with tight binding are located within the binding groove, while the residues with weak binding are located close to the binding site for the N-terminal tyrosine of the ligand, which is not part of the natural substrate and is therefore presumably bound with less restrictive restraints to the protein surface. The remaining residues of the protein have fitted affinities close to the average. It would therefore appear that residues located “in the binding site” of this rigid protein bind to the ligand more tightly than residues elsewhere. Weakly binding residues can be rationalised as being affected by motion of the N-terminal tyrosine, while the more distant residues sense an “average” affinity.

Barnase has a well-characterised binding site for this ligand, as illustrated in Fig. 6c. Residues with extreme affinities are not localised around the binding site as clearly as seen for SH3b, but there is nonetheless a tendency for strong affinities to be observed for residues in contact with the ligand, and weak affinities for more distant residues (Fig. 6c, d). The lack of definition by comparison to SH3b is not a consequence of errors in the data, which have been checked extensively.

HisJ has a well-characterised binding site for histidine, shown in Fig. 6e, f. The binding site for lysine has been shown to be in a similar location (Paul et al. 2017). The protein undergoes a major conformational change on binding, and closes around the ligand, with a hinge that bends to enclose the substrate inside the protein. For this binding interaction, there is no discernible relationship between *K*_d_ and proximity to the ligand (Fig. 6e, f). For example, there are 12 amino acid residues within 4 Å of the histidine ligand in the complex. Of these, 4 have a significantly small *K*_d_ and one has a large *K*_d_: Y17^N^, Y17^H^, R80^H^ and S73^H^ have small values with *K*_d_ of 53.9, 44.5, 40.9 and 38.6 μM respectively, while T124^N^ and T124^H^ have large *K*_d_ of 70.4 and 63.7 μM respectively. These ratios are not significantly different from random, in that 4 small and one large out of 12 that are close to the histidine ligand are not statistically different from the 33 small and 28 large out of 119 values fitted for the entire protein.

Thus in summary, for SH3b there is a clear and readily rationalised relationship between *K*_d_ and proximity to the ligand, with strong affinities in the binding groove and weak affinities close to the extra N-terminal tyrosine. For barnase this relationship is less marked but still evident, with residues that directly contact the ligand tending to have a stronger affinity, while for HisJ there is no clear relationship at all. These observations parallel the nature of the structural effects on the protein of ligand binding: SH3b has only minimal structural change on binding, barnase has a small amount of “induced fit” closing on binding, while HisJ has a major conformational closure.

## Discussion

### Affinities should be obtained by fitting all shift changes together

The results detailed in Table 1 make it clear that the most precise values for affinities are obtained by fitting all ^1^H and ^15^N shift changes together to a single affinity. The practice of combining ^1^H and ^15^N shift changes together (Eq. 2) is useful for graphic presentation of residue-specific changes, but is not useful for calculating affinities. This conclusion fits with conventional wisdom that the error in the fitted value goes down as $1/\sqrt{n}$, implying that it is important to maximise *n* by using all nuclei separately. It is usually not clear how published affinities are calculated, but one suspects that it is often done by picking a small number of signals with large shifts and using these. The results presented here demonstrate that this is not best practice, in that it leads to a larger standard error of the mean, and is thus less precise. It also leads to a modest increase (weakening) of the fitted dissociation constant, so is also less accurate. We note that there are several programs already available that can effectively be used to carry out such an analysis, including SEDPHAT (https://sedfitsedphat.github.io/sedphat/default.htm) which can accept a wide range of experimental data as input. We also note the program TITAN (Waudby et al. 2016), which is mainly designed for lineshape fitting but could also carry out such an analysis. Because of TITAN’s ability to incorporate lineshape analysis, it can be used for intermediate exchange timescales, which are not well fitted using the methodology described here.

The fitting described here shows that the commonly used value for α, the parameter used to weight ^15^N shift changes relative to ^1^H shift changes, is protein-dependent, but that the currently most commonly used value of 0.14 is a reasonable compromise, at least for the three proteins studied here.

### Interpretation of variation in binding affinity

Binding affinity of proteins can be measured by a wide range of techniques, including changes in UV/Vis or fluorescence, radioligand assays, isothermal titration calorimetry, surface plasmon resonance, and microscale thermophoresis (Ma et al. 2018). All of these, except NMR, produce a single averaged value for the dissociation constant, except in the small number of cases where two distinctly different affinities can be measured for one ligand binding to a protein in two different locations. By contrast, NMR can yield a different fitted affinity for each residue. However, to date there are very few reports of more than one fitted affinity (see Tossavainen et al. 2018 for a rare example). This is not surprising: it is reasonably clear what a binding affinity means, but what does it mean to have a range of different affinities in different places on the same protein?

**Fig. 7:**
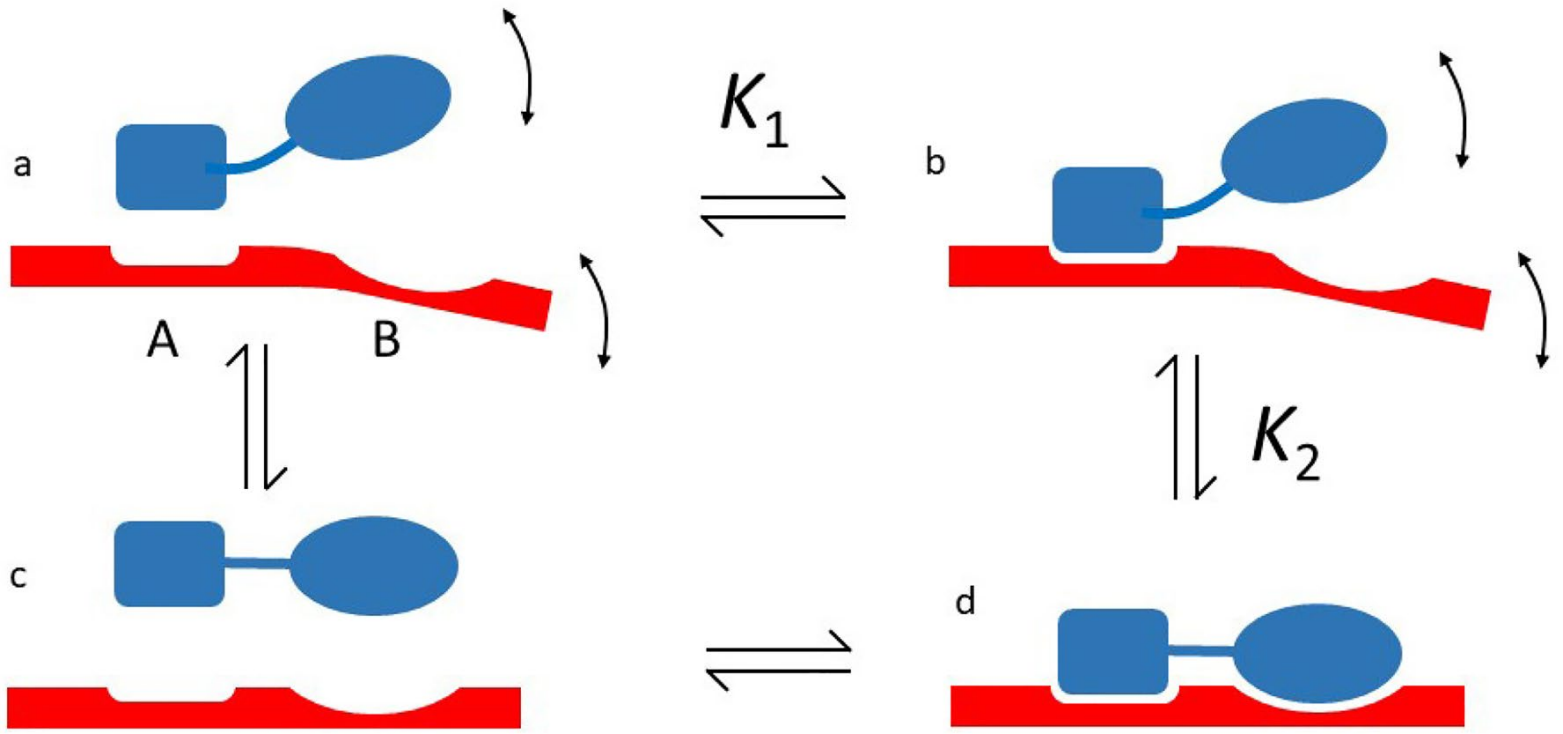
A simple model for flexible protein/ligand binding. Both the protein and the ligand may have internal flexibility (**a**). When they bind, the resulting complex has lost this flexibility (**d**). The process of binding may thus be separated conceptually into a rigid docking of part of the ligand (**a**, **b**), with an affinity *K*_1_, followed by an induced-fit type rearrangement accompanied by loss of flexibility (**b**–**d**). The equilibrium constant for this process is *K*_2_, which has values between 0 and 1, but may typically be expected to be greater than 0.5. Alternatively, the binding can be modelled as a loss of internal degrees of freedom (**a**–**c**) followed by a rigid docking (**c**, **d**)

A simple model is proposed in Fig. 7. If both protein and ligand are completely rigid (Fig. 7c, d), then it is a good approximation to say that the ligand can be either bound or free: no intermediate positions are available. However, if either the protein or the ligand has some flexibility, then we can represent the binding as a rigid lock-and-key docking for part of the ligand (Fig. 7a, b, binding constant *K*_1_), followed by a more flexible induced fit elsewhere (Fig. 7b–d). The induced fit rearrangements can be summed up by an equilibrium constant *K*_2_, which contains mainly losses of conformational entropy on rigidification (Fig. 7a–c) and gains of binding enthalpy on rigid binding at site B, and so is linked to concepts such as effective concentration (see for example Jencks 1975, Fersht 1999 and Williamson 2011 for further discussion). If parts of the ligand can be unbound while other parts are bound, then different affinities will be expected: the rigidly interacting parts will have an affinity *K*_1_ while the rest of the protein will have a weaker affinity *K* given by *K*_1_ × *K*_2_. On this model, residues displaying a tighter than average affinity are those residues that form a rigid docking site for the ligand, while the residues showing “average” affinity display some flexibility in their docking. The ratio between the tight and average affinity can be treated as a linkage parameter describing the local variability in the binding site geometry, providing a measure of the flexibility of the protein/ligand complex in the vicinity of the binding site. For the three proteins studied here, this parameter has a value of between 0.4 and 0.7. We note that the affinity characterised by the other methods mentioned at the start of this section corresponds to the average affinity measured by NMR. The tight affinity seen in NMR represents the affinity of rigidly frozen protein and ligand, which is neither feasible nor desirable, because it would dramatically slow down binding and release (Williamson 2011). The increase in on-rate and off-rate resulting from local flexibility is also proposed as one of the key advantages of fuzzy binding (Olsen et al. 2017), which is a more general version of the model proposed here.

This model works well for SH3b, where the fitting of the pentaglycine fragment into the groove provides a tight and inflexible docking, while surrounding residues are more able to adapt to the bound peptide, in particular to bind the more flexible N-terminal tyrosine of the ligand. Similar comments hold for barnase, where the tight binders form a directly contacting and relatively rigid platform for the ligand (Pandya et al. 2018). The large scale structural rearrangements of HisJ on binding mean that no such rigid binding platform can be identified for HisJ.

The pattern of variation of *K*_d_ provides an experimental measure of conformational flexibility on binding, with the very localised variation seen for SH3b (Fig. 6a, b) providing a clear indication of a rigid docking site, while the much more widespread changes seen for HisJ imply a conformational change distributed around the protein structure. To be a useful guide, it is of importance to obtain accurate fits of affinities for individual signals. We note that there are a range of statistical techniques that can be applied to improve the accuracy of fitting, of which one that has been applied with some success is singular value decomposition, which can significantly reduce the experimental noise associated with peak picking (Arai et al. 2012; Eaton and Williamson 2017).

## Supplementary Information

Below is the link to the electronic supplementary material.

Supplementary file1 (PDF 805 KB)

Supplementary file2 (XLSX 32 KB)

Supplementary file3 (XLSX 266 KB)
