# Supplementary material for: The measurement of binding affinities by NMR chemical shift perturbation
Source: J Biomol NMR. 2022 Aug 3;76(4):153–63. doi: 10.1007/s10858-022-00402-3 (PMC9427925; doi:10.1007/s10858-022-00402-3)
Supplement: Supplementary file 1 — Supplementary file1 (PDF 805 KB) [file 10858_2022_402_MOESM1_ESM.pdf]

The measurement of binding affinities by NMR chemical shift perturbation

Billy Hobbs, Jack Drant, Mike P Williamson

Supplementary Information

The SI consists of plots of fitted K_d values for barnase and SH3b, before and after filtering of the data, corresponding to the data shown for HisJ in Figs. 4 and 5. In separate Excel files we provide the raw chemical shift data from the titrations together with fitted values, including residues considered to have K_d values significantly larger or smaller than the mean for each protein.

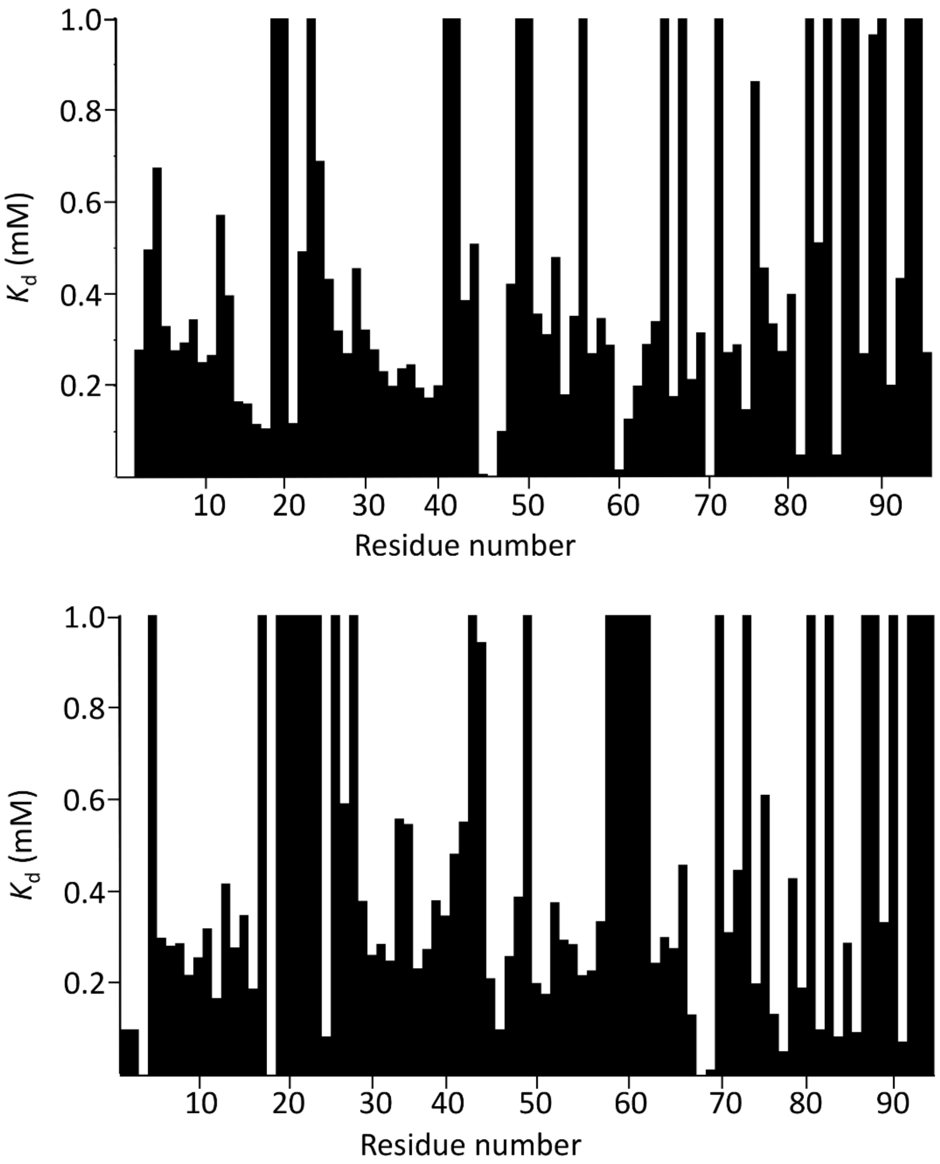

Fig. S1 K_d values fitted for SH3b binding to YG_5, for (top) ^1H (bottom) ^{15}N. Data are shown for all residues that could be fitted. A small number are not shown, mainly because of overlap or because they are prolines. Note that the K_d values are truncated at 1.0 mM: many are much larger than this.

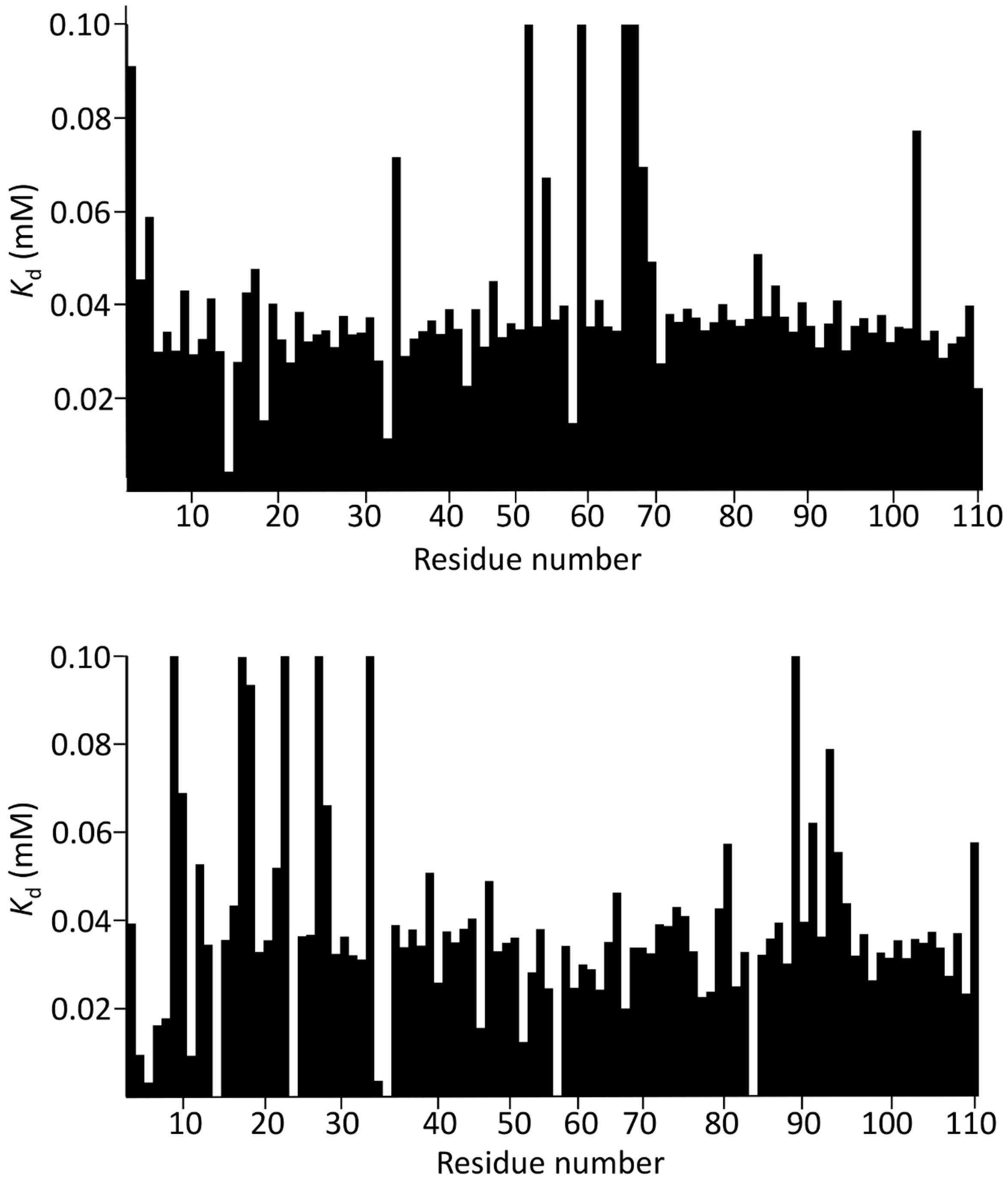

Fig. S2 K_d values fitted for barnase binding to d(CGAC), for (top) ^1H (bottom) ^{15}N. Data are shown for all residues that could be fitted. A small number are not shown, mainly because of overlap. Note that the K_d values are truncated at 0.1 mM: many are much larger than this.

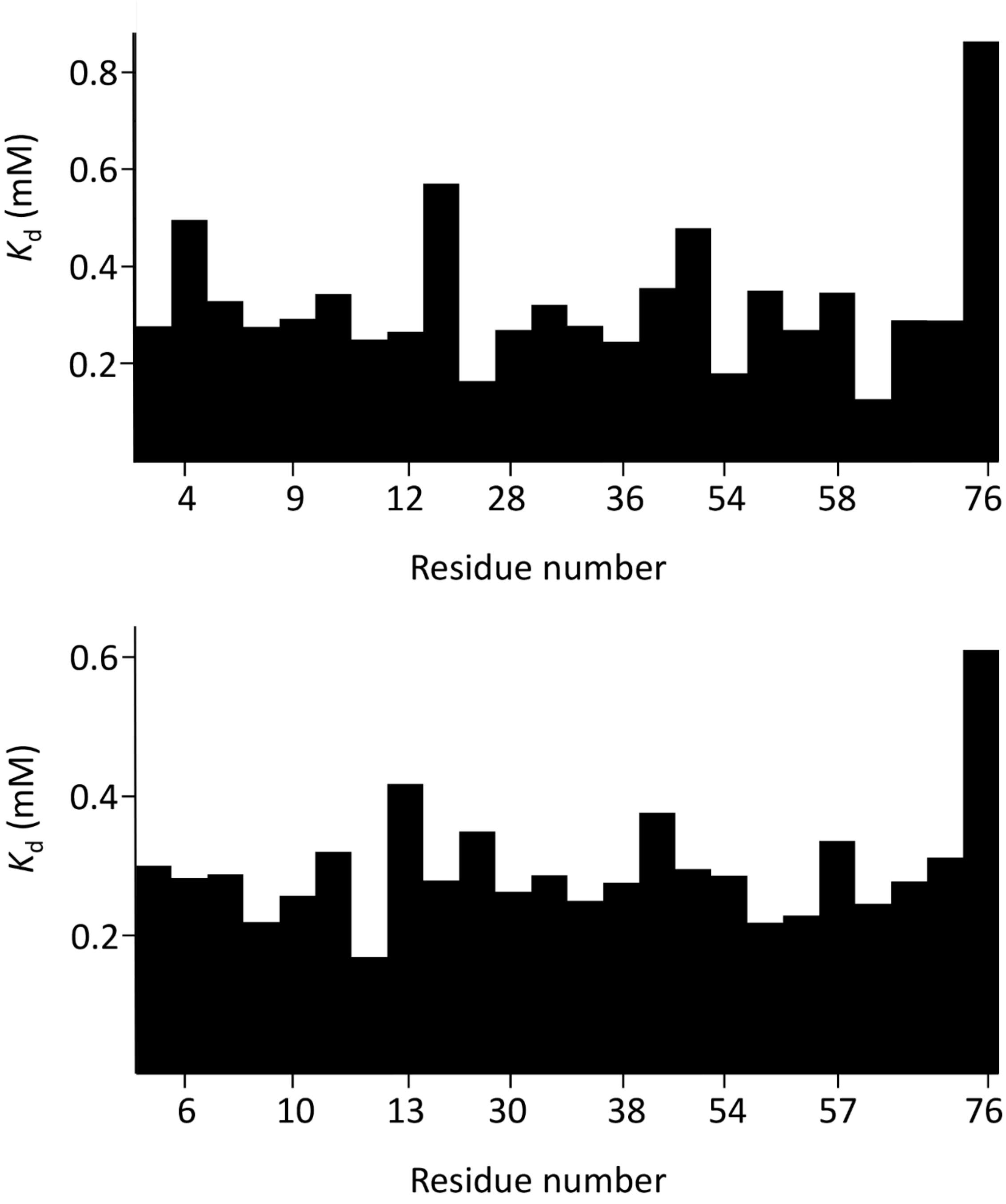

Fig. S3 K_d values fitted for SH3b binding to YG_5, after filtering out unreliable values, for (top) ^{1}H (bottom) ^{15}N. Nuclei were removed if they had a fitted K_d of > 1 mM, total ^{1}H shift changes of < 0.03 ppm, and total ^{15}N changes of < 0.15 ppm.

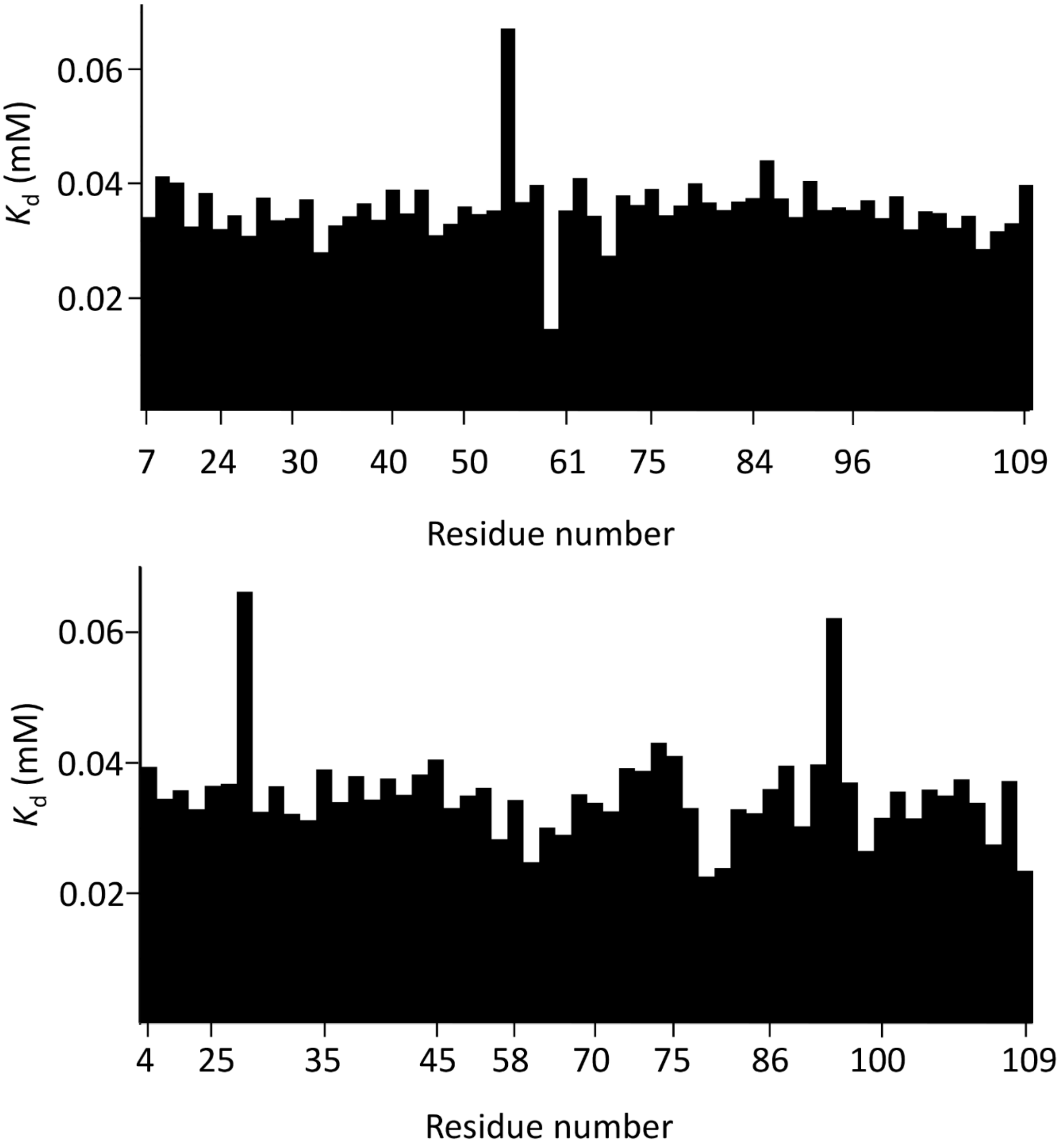

Fig. S4 K_d values fitted for barnase binding to d(CGAC), after filtering out unreliable values, for (top) ^{1}H (bottom) ^{15}N. Nuclei were removed if they had a fitted K_d of > 1 mM, total ^{1}H shift changes of < 0.025 ppm, and total ^{15}N changes of < 0.1 ppm.
